# The Influence of Provaping “Gatewatchers” on the Dissemination of COVID-19 Misinformation on Twitter: Analysis of Twitter Discourse Regarding Nicotine and the COVID-19 Pandemic

**DOI:** 10.2196/40331

**Published:** 2022-09-22

**Authors:** Nathan Silver, Elexis Kierstead, Ganna Kostygina, Hy Tran, Jodie Briggs, Sherry Emery, Barbara Schillo

**Affiliations:** 1 Schroeder Institute Truth Initiative Washington, DC United States; 2 Social Data Collaboratory NORC at the University of Chicago Chicago, IL United States

**Keywords:** social media, tobacco, COVID-19, nicotine, misinformation, Twitter, information, infodemiology, vaping, therapeutic, influence, environment, harmful, consequences

## Abstract

**Background:**

There is a lot of misinformation about a potential protective role of nicotine against COVID-19 spread on Twitter despite significant evidence to the contrary. We need to examine the role of vape advocates in the dissemination of such information through the lens of the gatewatching framework, which posits that top users can amplify and exert a disproportionate influence over the dissemination of certain content through curating, sharing, or, in the case of Twitter, retweeting it, serving more as a vector for misinformation rather than the source.

**Objective:**

This research examines the Twitter discourse at the intersection of COVID-19 and tobacco (1) to identify the extent to which the most outspoken contributors to this conversation self-identify as vaping advocates and (2) to understand how and to what extent these vape advocates serve as gatewatchers through disseminating content about a therapeutic role of tobacco, nicotine, or vaping against COVID-19.

**Methods:**

Tweets about tobacco, nicotine, or vaping and COVID-19 (N=1,420,271) posted during the first 9 months of the pandemic (January-September 2020) were identified from within a larger corpus of tobacco-related tweets using validated keyword filters. The top posters (ie, tweeters and retweeters) were identified and characterized, along with the most shared Uniform Resource Locators (URLs), most used hashtags, and the 1000 most retweeted posts. Finally, we examined the role of both top users and vape advocates in retweeting the most retweeted posts about the therapeutic role of nicotine, tobacco, or vaping against COVID-19.

**Results:**

Vape advocates comprised between 49.7% (n=81) of top 163 and 88% (n=22) of top 25 users discussing COVID-19 and tobacco on Twitter. Content about the ability of tobacco, nicotine, or vaping to treat or prevent COVID-19 was disseminated broadly, accounting for 22.5% (n=57) of the most shared URLs and 10% (n=107) of the most retweeted tweets. Finally, among top users, retweets comprised an average of 78.6% of the posts from vape advocates compared to 53.1% from others (*z*=3.34, *P*<.001). Vape advocates were also more likely to retweet the top tweeted posts about a therapeutic role of nicotine, with 63% (n=51) of vape advocates retweeting at least 1 post compared to 40.3% (n=29) of other top users (*z*=2.80, *P*=.01).

**Conclusions:**

Provaping users dominated discussions of tobacco use during the COVID-19 pandemic on Twitter and were instrumental in disseminating the most retweeted posts about a potential therapeutic role of tobacco use against the virus. Subsequent research is needed to better understand the extent of this influence and how to mitigate the influence of vape advocates over the broader narrative of tobacco regulation on Twitter.

## Introduction

### Background

During health crises, such as the COVID-19 pandemic, people who are at higher risk of being affected may be more likely to seek health information online [1,2]. However, in the absence of clear, concise, and complete information, digital information channels, such as social media, are often used to help people understand the implications of a health threat [3,4]. Since the use of inhaled nicotine products, such as cigarettes and e-cigarettes, enhances the risk of respiratory illness and places users at greater risk of complications from COVID-19, clear communication about the risks of tobacco use is more important than ever [5-8]. Some have suggested that a widespread respiratory virus, such as COVID-19, could provide an opportunity to amplify public perceptions about the harms of tobacco products [9]. However, conflicting claims about how the virus affected tobacco users quickly emerged [10,11].

A review of early clinical data in Wuhan Province found that smokers were less likely to be admitted to the intensive care unit (ICU) due to COVID-19 complications compared to nonsmokers [10]. Although the study itself did not provide any evidence of a causal relationship between smoking and COVID-19 infection or progression, the authors posited that the anti-inflammatory properties of nicotine might be responsible for the unexpectedly low prevalence of COVID-19–infected smokers in countries with high smoking rates [12,13]. Although no subsequent evidence has been found to support a protective role of nicotine, the notion that smoking, vaping, or nicotine use would prevent COVID-19 circulated, leading researchers to document misinformation about smoking, vaping, and nicotine as being protective against COVID-19 across communication channels, particularly on Twitter [14-16]. Although the spread of problematic information is not unique to Twitter, recent survey data suggest that Twitter users in particular are more likely to recall hearing and believe that nicotine, tobacco, or vaping can prevent COVID-19 [17-20].

The presentation of scientific findings from an early review of clinical records showing fewer smokers than expected among ICU patients as evidence that nicotine prevents COVID-19 is emblematic of the role social media often plays in communications from the scientific establishment to the general public [14]. Such counterintuitive findings are not misinformation in a direct sense, in that they do not present demonstrably false information [21]. Rather, the extrapolation of the study’s findings out of context or with overreaching implications exemplifies the sort of claims that are not egregiously false but rather represent unsubstantiated and misleading implications that run counter to the best-available scientific evidence [11,22].

To understand how scientific distortions and misinformation spread on Twitter, it is first important to understand key differences in how traditional news outlets and social media sites disseminate content. Media researchers use variations of the “gates” metaphor to describe how and to what extent elites and other opinion leaders dictate what information passes “through the gates” and on to the masses [23-26]. The first important difference between traditional news media, such as television, print, or even online publications and Twitter, is the elimination of “gatekeepers”—editorial boards and elite decision makers who determine which news receives airtime [27]. However, Twitter’s lack of traditional gatekeepers does not mean that the gates controlling the flow of information are left untended. Rather, the most influential users serve as “gatewatchers,” who lack absolute control over what passes through the gates but instead heavily influence whether information is channeled into high-traffic areas where it is likely to spread or low-traffic areas where its impact is diluted [28,29]. Thus, rather than a simple 2-step flow of news through a small group of elites to the viewing public, the preferences and ideological lean of a slightly larger group of vocal users dramatically influence what content “trends” in a user-driven marketplace of ideas [30,31].

Existing evidence suggests that the gatewatching framework may be useful to conceptualize how vaping and other tobacco-related information disseminates on Twitter. Previous research has found that social media discourse is predominantly hostile to vaping regulation, prone to exaggerated claims about the health benefits of vaping, and rife with misinformation about vaping and the tobacco industry [18,32-36]. Although the controversial nature of misinformation is often an important factor contributing to its spread, if the loudest and most prolific voices discussing vaping on Twitter are those with a provaping agenda, then such provaping gatewatchers are also a crucial pathway through which misinformation, disinformation, and other problematic or unsubstantiated information spreads on the medium.

Although there is evidence of a provaping bias on Twitter, neither the extent of this bias nor the influence on the volume of pro- versus antivaping content is clear. A recent examination of vaping-related tweets between March and June 2020 found that misinformation about the relationship between COVID-19 and vaping informed chatter that was both pro- as well as antivaping [37]. In a separate study, the same researchers showed that misinformation was endemic to the Twitter discourse about vaping even prior to COVID-19 [36]. Previous research examining the prevalence of the claim that nicotine can prevent COVID-19 found the therapeutic nicotine claim to be prevalent in about 1% of tweets relevant to both the pandemic and tobacco [16]. Building on these findings, we suggest that vape advocates who disproportionately influence the tobacco-related information that trends on Twitter (ie, gatewatchers) were likely instrumental in disseminating content that promoted a therapeutic role of tobacco, nicotine, or vaping against COVID-19.

### This Study

This research examines whether the gatewatching framework can be used to understand how content about a therapeutic benefit of nicotine, tobacco, or vaping disseminated on Twitter. The main premise of the gatewatching framework is that a subset of influential users drives the dissemination of information on Twitter through retweeting content that is consistent with their ideological agenda. Our investigation begins with the assumption that influence on Twitter is concentrated among a small group of top users who produce and disseminate the majority of content. Pew’s population-level examination of Twitter behavior supports this assumption [38]. Pew estimates that 97% of tweets are produced by the top 25% of users. Moreover, a high percentage of tweets by these top users are likely to be retweets of other users’ original tweets. The influence of provaping gatewatchers would thus be evident in (1) high prevalence of vape advocates among top users, (2) substantial dissemination of ideologically aligned content (eg, tobacco, nicotine, or vaping could prevent COVID-19), and (3) direct evidence of the role of top users and vape advocates in disseminating that content. We thus propose the following research questions (RQs):

- RQ1: How prevalent are vape advocates among the users who produce and disseminate the most content (ie, potential gatewatchers)?
- RQ2: How prevalent was content indicative of provaping advocacy in the broader conversation about tobacco and COVID-19, including (1) top hashtags, (2) top shared Uniform Resource Locators (URLs), and (3) the most retweeted tweets?
- RQ3: What role do top users and vape advocates play in disseminating top content (ie, top retweeted tweets about a therapeutic role of tobacco, nicotine, or vaping against COVID-19)?

## Methods

### Procedure

We began by identifying posts (original tweets and retweets) about COVID-19 from within the entire corpus of tobacco-related tweets posted between January and September 2020. After cleaning and preprocessing the raw data, including removing duplicate posts, we examined the data set at both the post and the user level. At the post level, we conducted a content analysis of the top 1000 retweets during this period to assess the volume of broadly disseminated tweets promoting the preventative nicotine claim compared to the 1% of overall tweets identified in previous research [16]. We then examined the most shared URLs to further quantify how much content promoting a therapeutic benefit of nicotine against COVID-19 was disseminated on Twitter. Next, we examined the user profiles of the most active users (ie, those responsible for the most tweets and retweets) and identified those who posted the original tweets about nicotine preventing COVID-19 among the top 1000 retweets. Finally, we cross-referenced the top user list with those who retweeted original tweets about nicotine preventing COVID-19 to more clearly illuminate the role of these top users in disseminating this content (ie, gatewatching).

### Data Collection

NORC at the University of Chicago maintains a comprehensive archive of tobacco-related Twitter data collected monthly using the Historical Powertrack Application Programming Interface (API) and sorted for relevance by a naive Bayes classifier. Twitter’s API allows for targeted searches by keywords that can appear in either the text of the tweet or the metadata. NORC “tapped the firehose” collecting all tweets posted during the study time frame in JSON format and then parsed and merged the data into a data frame at the post level with corresponding variables for username and other relevant metadata. From this broader corpus of tobacco-related tweets posted in the first 9 months of 2020, we developed and validated a keyword filter (Multimedia Appendix 1) to identify tobacco-related posts that were also about COVID-19. We then validated this filter by human-coding a random sample of 2566 original tweets for relevance (precision=0.90, recall=0.89, F_1_=.89). The text of each tweet was then used to extract important information, including URLs, hashtags, and whether it was an original tweet or a retweet. Counts were then aggregated to provide data frames at the post (tweet or retweet; N=1,420,271), user (N=817,691), and URL (N=54,806) levels and the top 1000 hashtags.

#### Identifying Top Users

We first sought to identify a smaller group of top users who were clear outliers in terms of the proportion of overall tweets and retweets for which they were responsible. We began with the top 1000 users who posted (both original and retweeted) between 54 and 4897 times each, meaning 0.12% of users were directly (tweeted) or indirectly (retweeted) responsible for 10.93% of all tobacco and COVID-19 content. Among these top 1000, we identified 2 natural inflection points in the data: (1) The median number of posts was 87 (SD 263.10). Only 25 (2.5%) users posted 3 or more SDs from the median number of posts, meaning 2.49% of all activity came from the top 25 users. (2) To expand this list further, we subgrouped the number of tweets per user in bins of 100, with 59% having less than 100 tweets and 83% having less than 200. We thus coded 163 top users who had 200 or more posts and were responsible for 5.59% of all content produced in our data set. Our coded sample of the most influential users averaged 54.15 tweets per month, more than double the threshold for high-volume users set by Pew. We then categorized these top 163 users by identifying at least 1 of 3 criteria in their profiles: (1) explicit mention of vaping or tobacco harm reduction (THR) in the text of the username or profile, (2) a pinned tweet (a tweet that the user chooses to fix to the top of their page) promoting vaping, or (3) at least 3 of their 5 most recent tweets explicitly promoting vaping.

#### Identifying Dissemination of Therapeutic Nicotine Content

We examined 3 key measures of trending content for dissemination of misinformation related to a potential therapeutic role of nicotine against COVID-19. First, we examined the top trending hashtags during this period. Hashtags are a key means through which social media conversations coalesce around a coherent narrative [39,40]. All hashtags were extracted from the text of the tweets and aggregated. Beginning with the top 1000 hashtags that were used between 50 (#heart) and 87,566 times (#covid19) with a median of 106.50 (SD 3101.53), we identified a natural inflection point in the data wherein only 16 hashtags were used greater than 1 SD from the median, accounting for 49.3% of hashtags used. We then identified hashtags that were explicitly tied to vaping, e-cigarettes, or tobacco harm reduction using keyword stems (eg, vap*, ecig*, thr, and harmreduc*).

Top linked URLs were examined using a similar procedure in finding a natural inflection point in the data to determine the top trending content. URLs were shared a median of 1 time each (SD 24). Of those, 253 URLs were shared greater than 3 SD from the median, comprising 30.9% of all shared URLs. The number of shares for these top URLs ranged from 74 to 2827, with a median of 117.5 (SD 279.73). We then examined these top URLs to determine whether they were linking content that promoted the ability of nicotine, tobacco, or vaping to prevent or treat COVID-19.

Finally, we conducted a content analysis of the top 1000 retweets to characterize the presence of original tweets about a potential therapeutic benefit of nicotine, tobacco, or vaping against COVID-19 in the most broadly disseminated part of the broader conversation about tobacco and COVID-19 on Twitter. We used a grounded theory approach [41,42]. We reviewed the top 1000 retweets while noting 6 relevant themes, with the primary theme of interest being the potential therapeutic role of nicotine or tobacco against COVID-19. Consistent with the convention for content analyses, a random subsample of at least 10% was withheld to establish reliability [43]. In this study, 2 independent coders dual coded a random subsample of 300 (30%) retweets to establish reliability in identifying tweets about personal responsibility (κ=0.95), social justice (κ=0.83), discounting COVID-19 severity compared to tobacco (κ=1), government criticism (κ=0.92), mask efficacy (κ=0.8), and, the topic of interest, the protective role of nicotine (κ=1). After establishing reliability, the remaining 700 (70%) retweets were divided evenly among the coders. The user profiles of those whose retweets promoted a therapeutic role of nicotine against COVID-19 were then coded to identify vaping advocates using the same methodology as was used for coding the top users. Finally, our RQ about gatewatching was then examined by identifying whether the top users retweeted the top retweeted content about a potential therapeutic role of nicotine or tobacco.

#### Bot Detection

The role of automated (bot) accounts on Twitter has been a recent area of concern [44]. One report suggested that as many as half of all tweets about vaping may come from bots [44]. Although a bot programmed to promote vaping content serves functionally the same purpose as a human gatewatcher who promotes vaping content, differentiating between bots and human accounts is important, as regulatory bodies and health communicators are likely to approach these sources of problematic information in different ways [45]. We first used the machine learning classifier *Botometer* to estimate the likelihood that user accounts were bots based on a series of indicators of “botlike” behaviors identified by the tool’s creators, providing a score between 0 and 5, with 5 being the most likely to be a bot [46]. However, this tool has been shown to have significant limitations in misclassifying both bot and human accounts [47]. As a result, we reported additional indicators that may be indicative of bot activity, including whether the account is verified by Twitter and whether the account has since been removed or made private. It is noteworthy that the top user overall had a bot score of 3.6 and only posted original tweets. However, this user was not a vape advocate.

### Ethical Considerations

Human subjects were not involved in this study. Data were collected from public social media sites. Account names were excluded for anonymity in publication.

## Results

### Vape Advocates and Vaping Hashtags

Vape advocates were highly prevalent across the top users, representing 81 (49.7%) of the top 163 users and 22 (88%) of the top 25 users. These top 163 users posted a median of 317 times each (SD 536.56) and retweeted (median 234, SD 373.80) far more often than they posted original tweets (median 58, SD 433.98). On average, retweets comprised a higher percentage of posts for vape advocates (78.6%) than for others (53.1%; *z*=3.34, *P*<.001). The prevalence of bots among top users appeared limited. Although only 3 accounts were verified by Twitter and 20 were either removed or private, the average Botometer score was low (mean 1.59, SD 1.37), with only 19% (n=31) of users having above the scale’s midpoint of 2.5. The average score for vape advocates was 1.26 but 2.03 for all other accounts, providing little evidence that bots are driving vape advocacy in our data set.

Vaping hashtags (n=63) were used a total of 43,223 times, accounting for 9.4% of the hashtags used in our data set, including 3 of the top 16 most used overall. Table 1 provides the top 16 overall hashtags in the data set as well as the top 16 vaping hashtags, accounting for 85.7% of the vaping hashtags used. Most noteworthy is the use of #wevapewevote among the top overall hashtags as well as 5 other explicitly provaping hashtags, with over 1000 uses each.

Of the 253 top shared URLs, 57 (22.5%) promoted content about a potential therapeutic role of nicotine, tobacco, or vaping in treating or preventing COVID-19. These URLs were shared 16,244 times. Table 2 provides descriptions of the top 29 shared URLs identified via an inflection point in the data at more than 2 SD from the median number of shares, accounting for 12.4% of all shared URLs. Among these top 29 URLs, 12 URLs linked articles promoting the potential therapeutic value of nicotine, vaping, or tobacco against COVID-19, accounting for 41.4% of shares among this top content. It is noteworthy that 2 (17%) of these 12 were articles explicitly debunking the claim made by a media personality that vaping bleach could cure COVID-19, while the other 10 (83%) focused on either a lower infection rate of COVID-19 for smokers (n=9, 90%) or a tobacco-based vaccine (n=1, 10%).

The top 1000 retweeted posts were shared a total of 578,763 times, ranging between 105 and 117,662, with a median of 193 (SD 3956.82). Table 3 provides the 6 coded categories, example tweets, and the percentage of retweets in each category. The therapeutic potential of nicotine or tobacco was the fourth-most commonly discussed topic. Of the 107 retweeted posts addressing the protective role of nicotine, including smoking, vaping, or tobacco in general, 5 (4.7%) sought to counter this notion and accounted for 1304 (0.2%) retweets. Closer examination also revealed that 4 of these retweets (3.7%) concerned addressing a conservative talk show host who told a call-in listener that they could vape bleach to protect themselves from COVID-19. After removing these 9 (8.4%) tweets, we focused on 98 (91.6%) of the top retweets explicitly endorsing or promoting the idea that nicotine, whether through patches, smoking, or vaping, could prevent COVID-19. Such content was retweeted 21,782 times, garnering 3.8% of retweets in our sample (median 160, SD 194.25). Moreover, these tweets also used the hashtags #saysscience (n=17, 17%) and #sciencesurprises (n=12, 12%), which were used across 2129 and 1544 retweets, respectively.

A total of 74 unique users produced the 98 top retweets about a therapeutic role of nicotine. Of these, 30 (40.5%) were verified, while 16 (21.6%) were official news accounts. In fact, retweets by verified accounts garnered 74.5% of retweets, while news accounts (all but 2 of which were verified) garnered 46.2% of retweets. There were only 2 (2.7%) vape advocates among these 74 users. Finally, bots were limited among this group as well, with an average Botometer score of 1.57 (SD 1.28). Notably, this value is likely inflated as the verified news sources tended to be misclassified as bots, with an average score of 3.19.

The top 163 users retweeted the top posts about nicotine preventing COVID-19 338 times (median 1, SD 2.78). Among the top 163 users, 91 (55.8%) retweeted at least 1 of the top posts, with 17 (68%) of the top 25 retweeting at least 1 post (median 4, SD 4.51). A significantly higher percentage of vape advocates (63%) retweeted such posts compared to other top users (40.3%; z=2.80, *P*=.01). In total, 38.2% of top posts were retweeted at least once by top users, with original posts by the lead author of the study showing a lower-than-expected number of smokers in the ICU with COVID-19 garnering 38.5% of retweets by top users. Table 4 provides the deidentified text of the top retweets promoting such content retweeted by top users.

**Table 1:** Top hashtags for COVID-19– and nicotine-related discussions on Twitter.

Top 16 overall hashtags^a^ (N=87,566)		Top 16 vaping hashtags^b^ (N=43,223)	
Hashtags	Uses, n (%)	Hashtags	Uses, n (%)
covid19	87,566 (100)	vaping^c^	15,567 (36.0)
coronavirus	31,608 (36.1)	vape^c^	6306 (14.6)
vaping	15,567 (17.8)	wevapewevote	3601 (8.3)
nomeat_nocoronavirus	11,495 (13.1)	vapingsaveslives	2419 (5.6)
tobacco	10,961 (12.5)	ecigs^c^	1495 (3.5)
covid-19	9181 (10.5)	vapefam	1283 (3.0)
smoking	8328 (9.5)	harmreduction	1137 (2.6)
covid	8193 (9.4)	vapers	1137 (2.6)
lockdownsa	7685 (8.8)	ecigarettes^c^	871 (2.0)
covid_19	7256 (8.3)	ecig^c^	766 (1.8)
stayhome	6705 (7.7)	vapelife	734 (1.7)
vape	6306 (7.2)	vapes	502 (1.2)
quitforcovid	5156 (5.9)	tobaccoharmreduction	424 (1.0)
lockdown	4021 (4.6)	vapeon	414 (1.0)
wevapewevote	3601 (4.1)	vapecommunity	390 (0.9)
indiafightscorona	3472 (4.0)	vapenation	387 (0.9)

^a^The top 16 hashtags accounted for 49.3% of all hashtags used.

^b^The top 16 vaping hashtags accounted for 85.71% of all provaping hashtags used and 9.4% of all hashtags used.

^c^These hashtags were not necessarily provaping and were sometimes used in antivaping posts as well.

**Table 2:** Top 29^a^ URLs^b^ shared in COVID-19– and nicotine-related discussions on Twitter.

URL description	Shares (N=21,100), n (%)
News24.com:lockdown dlamini zuma pushes for tobacco alcohol ban to continue until level 1	2827 (13.4)
The economist: smokers seem less likely than non smokers to fall ill with covid 19	2359 (11.2)
News24.com: coronavirus all the latest news about covid 19 in south africa and the world	1854 (8.8)
Raw story: conservative radio host agrees with caller that vaping bleach might cure covid 19 youre not crazy	878 (4.2)
The Guardian: French study suggests smokers at lower risk of getting coronavirus	796 (3.8)
News24.com: breaking ramaphosa told to lift cigarette alcohol ban and move to level 2 lockdown sources	694 (3.3)
rFi.fr: french researchers suggest nicotine could protect against covid 19	677 (3.2)
France24.com: france testing whether nicotine could prevent coronavirus 1	667 (3.2)
CNN: coronavirus quitting smoking wellness	657 (3.1)
News 24: not selling booze and tobacco during lockdown harmful to addicts	649 (3.1)
ewn.co.za: sa economy loses r1 5 billion due to alcohol cigarette sale ban	631 (3.0)
Telegraph: smokers four times less likely contract covid 19 prompting nicotine/	620 (2.9)
News24.com: coronavirus all the latest news about covid 19 in south africa and the world	584 (2.8)
Livemint.com: cigarette can keep coronavirus away researchers test if nicotine could prevent covid 19	557 (2.6)
Media Matters: Sean Hannity suggests vaping prevents people getting coronavirus	491 (2.3)
Money Control: a cigarette a day can keep coronavirus away french researchers test if nicotine can prevent covid	488 (2.3)
Bloomberg: coronavirus vaccine race gets unlikely partner big tobacco	472 (2.2)
W24.co.za: How lockdown saved my life woman shares how she finally quit smoking after 20 years	468 (2.2)
Scientific American: Smoking or vaping may increase the risk of a severe coronavirus infection	461 (2.2)
CNN: coronavirus quitting smoking wellness	461 (2.2)
The Guardian: french study suggests smokers at lower risk of getting coronavirus	457 (2.2)
NDTV: coronavirus drug news france testing if nicotine prevents coronavirus from attaching to cells	444 (2.1)
Nature: Factors associated with COVID-19-related death using OpenSAFELY	435 (2.1)
The Guardian: Politics public covid 19 tobacco johnson	431 (2.0)
CNN: coronavirus quitting smoking wellness	423 (2.0)
Zerohedge.com: Did china steal coronavirus from canada and weaponize it	409 (1.9)
Buzzfeed: smoking doesnt kill and other great old op eds from mike pence	408 (1.9)
Medium: how i killed the smoke monster and quit smoking like a queen	402 (1.9)
Todayistheday.co.uk/ (Resources for smoking cessation)	400 (1.9)

^a^These 29 web articles accounted for 12.4% of shares among a total of 54,806 different URLs that were shared a combined total of 170,496 times.

^b^URL: Uniform Resource Locator.

**Table 3:** Content analysis of the top retweets (N=1000)^a^ about COVID-19 and nicotine.

Coded category	Example	Top retweets, n (%)
Government criticism	MIKE PENCE: - His budget cuts in Indiana led to the spread of HIV there - Wrote articles about how smoking does NOT cause cancer - Calls global warming a myth - Was put in charge, by trump, of the Coronavirus response UNBELIEVABLE. #CoronaVirusUpdates	293 (29.3)
Personal responsibility	As COVID-19 attacks the lungs one of the most important things you can do is to quit smoking and vaping. I’m in day 3. Care to join?	149 (14.9)
Mask efficacy	Ok I recorded the recording because I know they will remove it... why?? Vape smoke is 2.5 microns... Covid is between 0.15-0.25 microns. Masks don’t do shit.	149 (14.9)
Protective role of nicotine	Nicotine could protect people from contracting the coronavirus, according to new research in France, where further trials are planned to test whether the substance could be used to prevent or treat the deadly illness	107 (10.7)
Discounting the pandemic’s impact	If they're going to report every Coronavirus death, I think they should have to report every: Flu Death Car Accident Death Smoking-related Death Alcohol Related Death . . . You get the point. ENOUGH WITH THE FEAR MONGERING	50 (5.0)
Social justice	imagine if the surgeon general announced a plan to bolster access to masks, testing, &; neighborhood health hubs for Black & Latinx people instead of telling us to not smoke &; drink to protect big momma'n'em. how do you blame people for being imperfect victims of a pandemic?	49 (4.9)

^a^Of the 1000 posts, 2 independent coders double-coded 300 (30%) posts to establish reliability, after which the remaining 700 (70%) were divided evenly between the 2 coders.

**Table 4:** Top 10 posts^a^ about nicotine preventing COVID-19 retweeted by top users.

Tweet text	Top users who retweeted, n (%)
NYC Mayor said smoking and vaping increases coronavirus risk. In 1099 cases from China, only 12.6% were smokers (we would expect much higher). ZERO data on e-cigs. So, still too early to say. People with ZERO public health knowledge should SHUT UP.	39 (23.9)
Finally, the study is out “Systematic review of the prevalence of current smoking among hospitalized COVID-19 patients in China could nicotine be a therapeutic option?” Very low prevalence of smoking among hospitalized COVID-19 patients in China.	38 (23.3)
Moderate and heavy smokers were 50-60% less likely to be tested positive for COVID-19 and 80-90% less likely to be admitted to the ICU... Remember my hypothesis about the potentially protective effects of nicotine since early April?	31 (19.0)
So few people hospitalized with the coronavirus appear to be smokers. I spoke to the scientists, tobacco experts, and policymakers who are trying to see if nicotine *might* have something to do with it.	23 (14.1)
Dramatic UNDER-representation of smokers among COVID-19 patients in France. 80% reduced standardized (for age and sex) incidence ratio!! Strongly supports my hypothesis about the protective effects of nicotine which i made 1 month ago (soon to be published).	22 (13.5)
On January 22 at the beginning of this year I had a suspicion about the protective effect of nicotine on the coronavirus.	20 (12.3)
Official French data on #tobacco smoking; #covid19 replicate the picture in China, Germany; USA A remarkable low rate of smokers are hospitalised w/ coronavirus compared to smoking prevalence (France 23%).	18 (11.0)
The government has admitted “smoking populations were less likely to be infected” with the coronavirus and develop Covid-19.	12 (7.4)
The prohibition of cigarettes does nothing to control or limit the Covid 19 epidemic. On the contrary smokers are significantly less likely to require hospitalization if they do become infected. NDZ is pursuing a very personal and subjective campaign.	12 (7.4)
“There is zero evidence that smoking will propagate or increase transmission of COVID-19.”—Dr Konstantinos Farsalinos, Cardiologist and anti-smoking researcher	11 (6.8)

^a^These top 10 posts accounted for 69.8% of retweets of such content by top users; 29 other posts were retweeted by between 1 and 9 top users.

## Discussion

### Principal Findings

This research demonstrates the utility of the gatewatching framework for examining the dissemination of problematic information on Twitter. More than half of our sample of top users and 22 of the 25 most prolific users producing and disseminating content about COVID-19 and tobacco in the first 9 months of the pandemic were provaping “harm reduction” advocates. Moreover, more than 3 of 4 posts by these top-using vape advocates were retweets compared to just over half for non-vape advocates, further demonstrating the key role of these users as disseminators of content—gatewatchers.

Building on previous research both identifying and quantifying the extent of a specific piece of misinformation that nicotine can prevent COVID-19, we showed the disproportionately broad reach of this claim across the most retweeted content during this period [16,37,48]. Even in May 2020, when the original study by Farsalinos et al [10] was published, the preponderance of scientific evidence, including multiple meta-analyses, still opposed the notion that nicotine, and especially smoking, would protect people from COVID-19. Still, in our sample of the top 1000 retweets, propagation of this claim was more than 20 times more common than the 5 tweets trying to debunk the claim and was retweeted nearly 17 times as often. Among the top shared URLs, articles promoting a potential therapeutic role of nicotine or tobacco accounted for nearly 1 in 3 shares. Explicitly provaping hashtags, such as #wevapewevote, #vapingsaveslives, and #vapefam, were abundant, indicating a significant representation of a provaping perspective across the broader COVID-19– and tobacco-related conversation.

Finally, this study provides compelling evidence that the top users, particularly vape advocates, were instrumental in disseminating the idea that nicotine can prevent COVID-19. Vape advocates were significantly more likely to retweet top tweets about a potential therapeutic role of nicotine. More than half of the top users retweeted at least 1 of the most retweeted tweets on the topic, while more than 1 in 3 of the most retweeted tweets was retweeted by at least 1 top user. These findings have implications for both tobacco control and the process of disseminating information on Twitter.

### Implications for Tobacco Control

The most important implication for tobacco control is that the dissemination of tobacco content on Twitter is heavily influenced by vape advocates. The extent to which COVID-19 served as further motivation for smokers or vapers trying to quit is uncertain. However, research examining this question has found mixed results at best [49-51]. Although the use of addictive substances during a pandemic is explained by far more variables than misinformation on social media, our findings suggest that the provaping ideological bias of the most prominent voices on Twitter engaging in the tobacco control conversation may help explain why misinformation promoting the protective role of nicotine was disseminated so broadly. Furthermore, previous research examining temporal trends in the tobacco sentiment on Twitter noted an increase in antitobacco sentiment in March 2020 at the beginning of the pandemic in the United States, followed by a rise in positive tobacco sentiment corresponding with the release of the preprint of the study showing fewer smokers than expected in Wuhan ICUs [52]. Our study shows that a likely driver of positive sentiment—that tobacco, nicotine, or vaping has therapeutic value against COVID-19—was spread frequently by influential vape advocates on the platform.

This research highlights the growing challenge of addressing scientific distortions that while not themselves misinformation can nonetheless drive false beliefs. There is no reason to believe that the study finding fewer smokers than expected was falsified. In fact, this “smoker’s paradox” drove significant research interest and calls to pre-register hypotheses toward the goal of rigorously investigating the effects of nicotine on COVID-19 [53]. A substantial body of literature has provided strong evidence that smoking during a respiratory pandemic increases the risk of severe illness and death [5-8]. Moreover, more detailed investigations of specific hypotheses surrounding a therapeutic effect of nicotine have revealed the opposite, as nicotine appears to aid the replication of SARS-CoV-2 rather than impede it [54]. Our study does not address this complicated body of literature. Rather, we show how an opportunistic overinterpretation of the findings of such a study can disseminate on Twitter through influential users for whom such findings support a broader narrative. The broader implication of these findings is that the dissemination of information about tobacco control on Twitter is subject to the interpretation of users who both strongly influence what information is disseminated and whose stated purpose on the platform is to oppose tobacco regulation.

### Implications for Understanding Misinformation Dissemination

It is important to note that our findings do not contradict previous work examining the prevalence of misinformation on the protective role of nicotine but rather add context that helps to characterize the process through which misinformation spreads on Twitter. Kavaluru et al [16] identified that the protective role of nicotine constitutes about 1% of the overall content, while Sidani et al [37] identified a variety of different misinformation claims that arose on Twitter about vaping products. Both studies provide an overview of the overall “firehose” of information. We sought to understand how a small percentage of the information from this firehose got diverted into the smaller and more influential pool of trending retweets. Although we do not discount the fundamental virality of controversy that previous research suggests can drive the dissemination of misinformation, we highlight the important utility of the “gatewatcher” metaphor in describing how misinformation disseminates on Twitter [55].

Opinion leaders on Twitter do not have control over the content posted on the platform. However, they have outsized influence over the dissemination of certain perspectives over others. Although more research is needed, we contribute strong evidence that the ideological lean of the most prolific tweeters on a given subject (provape users discussing COVID-19 and nicotine) directly influenced the spread of problematic information (that nicotine could prevent COVID-19) through retweeting much of the most broadly disseminated posts. These findings are reminiscent of previous research showing the majority of disinformation in another context, antivaccination, emanated from only 12 users [56]. However, in contrast to the “Disinformation Dozen,” provaping gatewatchers on Twitter do not produce and disseminate overtly false information. Rather, they serve as mediators between the scientific community and the broader, Twitter-using public, and privilege scientific findings that support a provaping narrative, while dismissing, ignoring, and countering a preponderance of evidence that does not.

### Implications for Practitioners

There are 2 useful implications of this research for practitioners. The first is that a small component of the overall conversation can have an outsized influence. Ultimately, most of the conversation about COVID-19 and nicotine was about COVID-19 and only tangentially mentioned nicotine or tobacco products. Although #wevapewevote was among the top trending hashtags, #nomeatnocoronavirus was over 3 times more prevalent. Moreover, 60% of our sample of top retweets involved criticizing the government, complaining about masks, and extolling the virtues of taking care of one’s body during the pandemic.

Social media’s unprecedented democratization of the fourth estate unfortunately nests genuine attempts to engage constructive public discourse alongside incoherent, misinformed, and often bad-faith commentary [57]. Trending hashtags and overall prevalence tell an important part of the story with regard to what and how information spreads on social media [58]. That controversy is interesting and that anybody can post anything with limited oversight are both established and intuitive reasons why misinformation is endemic to social media [59]. However, addressing such a problem requires a closer examination of the vectors through which some tweets spread while others do not. Misinformation about nicotine and COVID-19 does not comprise a majority or even a plurality of content about using nicotine products during the pandemic. However, misinformation about nicotine preventing COVID-19 circulated broadly most likely because it was consistent with the ideological agenda of the opinion leaders, gatewatchers, and most prolific tweeters on the subject.

The broader implication of this process of dissemination is that the proverbial deck is stacked against effective public health communication on Twitter. In the context of nicotine’s potential role in preventing COVID-19, Twitter undoubtedly amplified bad information when good information was available. The observable provaping bias of the most outspoken users discussing COVID-19 and nicotine inevitably meant that even attempts to debunk such information on the platform did not receive nearly the same amount of traffic. The most important implication is that this bias is likely to result in the continued prominence of the benefits of vaping, while underrepresenting and downplaying the harms.

### Limitations and Future Directions

The most important limitation of this research is related to the scope of our findings. We examined the influence of gatewatchers on 1 of many social media platforms, in the specific context of tobacco control and COVID-19 during the onset of the pandemic. The dissemination of misinformation on social media is likely to vary between different platforms, different contexts, and potentially within the broader context of tobacco control. Although our findings have generalizable implications, more research is needed to fully understand the interplay between platform, context, and specific kinds of mis- and disinformation, including distortions of scientific consensus.

A second limitation concerns the conclusiveness of our findings with regard to the central premise of the gatewatching framework—that top users were directly responsible for the broad dissemination of the most retweeted content. We provide conclusive evidence that the top users discussing COVID-19 in the context of tobacco, most of whom were vape advocates, retweeted many of the most broadly disseminated retweets about nicotine as potentially therapeutic against COVID-19. However, network approaches are needed in subsequent research to identify whether it is indeed the retweets by these vape advocates that catalyze the broad dissemination of this and other content. Moreover, whether the influence of vape advocates extends beyond the intersection of COVID-19 and tobacco into the broader discussion of tobacco regulation on Twitter and social media is an important topic for subsequent research.

Additionally, we analyzed a small fraction of a data set comprising nearly 1.5 million posts and hundreds of thousands of users. However, research examining misinformation networks online suggests that the best way to reduce misinformation is to identify and penalize the central nodes—the opinion leaders and gatewatchers who drive the virality of some information over others [60]. Although the sheer quantity of content produced is an admittedly blunt instrument for assessing influence, we show that the primary function of these top users, and to an even greater extent the vape advocates among these top users, is to amplify (ie, retweet) some original tweets over others. Subsequent research should adopt more sophisticated measures to assess sustained influence over tobacco regulatory discourse, as a whole, toward which intervention is most likely to be effective.

Finally, although our use of the API and extensive filtering from the broader tobacco-related discussion on Twitter is a strength of this study in providing a near census of relevant content, limitations related to the collection of data from private and removed accounts mean that there is inevitably some content we missed. We note that we were able to capture content from users whose accounts were made private after we collected data.

### Conclusion

The COVID-19 pandemic offered a potential opportunity to highlight the importance of respiratory health by underscoring the negative long-term consequences of inhaled nicotine product use. However, the ability of provaping opinion leaders or gatewatchers on Twitter to steer the narrative and promote misinformation about nicotine as protective against COVID-19 likely played a role in dampening any positive effect of the pandemic on tobacco use. Although anyone can post on Twitter, the makeup of Twitter’s tobacco and COVID-19 opinion leaders suggests that content about the dangers of tobacco and vaping does not spread with the same virality as messages that support the proliferation of vaping.

## Appendix

Multimedia Appendix 1 Top 30 search terms identifying COVID-19 tweets.

## Abbreviations

API: Application Programming Interface
ICU: intensive care unit
RQ: research question
URL: Uniform Resource Locator

## References

[1] Roberts M, Ehde D, Herring T, Alschuler K (2021). Public health adherence and information-seeking for people with chronic conditions during the early phase of the COVID-19 pandemic. PM R.

[2] Vanderpool RC, Huang GC, Mollica M, Gutierrez AI, Maynard CD (2021). Cancer information-seeking in an age of COVID-19: findings from the National Cancer Institute's Cancer Information Service. Health Commun.

[3] Neely S, Eldredge C, Sanders R (2021). Health information seeking behaviors on social media during the COVID-19 pandemic among American social networking site users: survey study. J Med Internet Res.

[4] Shearer E (2021). More Than Eight-in-Ten Americans Get News from Digital Devices.

[5] Alqahtani JS, Oyelade T, Aldhahir AM, Alghamdi SM, Almehmadi M, Alqahtani AS, Quaderi S, Mandal S, Hurst JR (2020). Prevalence, severity and mortality associated with COPD and smoking in patients with COVID-19: a rapid systematic review and meta-analysis. PLoS One.

[6] Gupta A, Nethan S, Mehrotra R (2021). Tobacco use as a well-recognized cause of severe COVID-19 manifestations. Respir Med.

[7] Patanavanich R, Glantz S (2020). Smoking is associated with COVID-19 progression: a meta-analysis. Nicotine Tob Res.

[8] Zhao Q, Meng M, Kumar R, Wu Y, Huang J, Lian N, Deng Y, Lin S (2020). The impact of COPD and smoking history on the severity of COVID-19: a systemic review and meta-analysis. J Med Virol.

[9] Popova L (2022). Carpe covid: using COVID-19 to communicate about harms of tobacco products. Tob Control.

[10] Farsalinos K, Barbouni A, Niaura R Smoking, vaping and hospitalization for COVID-19. Qeios.

[11] Majmundar A, Allem J, Cruz TB, Unger JB (2020). Public health concerns and unsubstantiated claims at the intersection of vaping and COVID-19. Nicotine Tob Res.

[12] Farsalinos K, Niaura R, Le Houezec J, Barbouni A, Tsatsakis A, Kouretas D, Vantarakis A, Poulas K (2020). Editorial: nicotine and SARS-CoV-2: COVID-19 may be a disease of the nicotinic cholinergic system. Toxicol Rep.

[13] ClinicalTrials (2021). Efficacy of Nicotine in Preventing COVID-19 Infection (NICOVID-PREV).

[14] Bandara NA, Herath J, Mehrnoush V (2021). Addressing e-cigarette health claims made on social media amidst the COVID-19 pandemic. World J Pediatr.

[15] Majmundar A, Allem J, Unger JB, Cruz TB (2021). Vaping and COVID-19: insights for public health and clinical care from Twitter. Int J Environ Res Public Health.

[16] Kavuluru R, Noh J, Rose S Twitter discourse on nicotine as potential prophylactic or therapeutic for COVID-19. medRxiv.

[17] Suarez-Lledo V, Alvarez-Galvez J (2021). Prevalence of health misinformation on social media: systematic review. J Med Internet Res.

[18] Tan AS, Bigman CA (2020). Misinformation about commercial tobacco products on social media—implications and research opportunities for reducing tobacco-related health disparities. Am J Public Health.

[19] Wang Y, McKee M, Torbica A, Stuckler D (2019). Systematic literature review on the spread of health-related misinformation on social media. Soc Sci Med.

[20] Silver NA, Kierstead EC, Briggs J, Schillo B (2022). Charming e-cigarette users with distorted science: a survey examining social media platform use, nicotine-related misinformation and attitudes towards the tobacco industry. BMJ Open.

[21] Vraga EK, Bode L (2020). Defining misinformation and understanding its bounded nature: using expertise and evidence for describing misinformation. Political Commun.

[22] Southwell BG, Brennen JSB, Paquin R, Boudewyns V, Zeng J (2022). Defining and measuring scientific misinformation. Ann Am Acad Pol Soc Sci.

[23] Bruns A (2011). Gatekeeping, gatewatching, real-time feedback: new challenges for journalism. BJR.

[24] Pearson GDH, Kosicki GM (2016). How way-finding is challenging gatekeeping in the digital age. Journal Stud.

[25] Shoemaker PJ, Timothy V (2009). Gatekeeping Theory.

[26] Vos T, Heinderyckx F (2015). Gatekeeping in Transition.

[27] Bro P, Wallberg F (2014). Digital gatekeeping. Digit Journal.

[28] Bruns A (2018). Gatewatching and news curation: journalism, social media, and the public sphere. Digital Formations, Volume 113.

[29] Bruns A, Highfield T (2015). Chapter 18: from news blogs to news on Twitter: gatewatching and collaborative news curation. Handbook of Digital Politics.

[30] Krittanawong C, Narasimhan B, Virk H, Narasimhan H, Hahn J, Wang Z (2020). Misinformation dissemination in Twitter in the COVID-19 era. Am J Med.

[31] Oyeyemi SO, Gabarron E, Wynn R (2014). Ebola, Twitter, and misinformation: a dangerous combination?. BMJ.

[32] Albarracin D, Shavitt S (2018). Attitudes and attitude change. Annu Rev Psychol.

[33] Kwon M, Park E (2020). Perceptions and sentiments about electronic cigarettes on social media platformsystematic review. JMIR Public Health Surveill.

[34] Lazard A, Saffer A, Wilcox G, Chung A, Mackert M, Bernhardt J (2016). E-cigarette social media messages: a text mining analysis of marketing and consumer conversations on Twitter. JMIR Public Health Surveill.

[35] McCausland K, Maycock B, Leaver T, Wolf K, Freeman B, Jancey J (2020). E-cigarette advocates on Twitter: content analysis of vaping-related tweets. JMIR Public Health Surveill.

[36] Sidani JE, Hoffman BL, Colditz JB, Melcher E, Taneja SB, Shensa A, Primack B, Davis E, Chu K (2022). E-cigarette-related nicotine misinformation on social media. Subst Use Misuse.

[37] Sidani JE, Hoffman B, Colditz JB, Wolynn R, Hsiao L, Chu K, Rose JJ, Shensa A, Davis E, Primack B (2022). Discussions and misinformation about electronic nicotine delivery systems and COVID-19: qualitative analysis of Twitter content. JMIR Form Res.

[38] Wojcik S, Hughes A (2019). Sizing Up Twitter Users.

[39] Enli G, Simonsen C (2017). ‘Social media logic’ meets professional norms: Twitter hashtags usage by journalists and politicians. Inf Commun Soc.

[40] Zappavigna M (2018). Searchable Talk: Hashtags and Social Media Metadiscourse.

[41] Chun Tie Y, Birks M, Francis K (2019). Grounded theory research: A design framework for novice researchers. SAGE Open Med.

[42] Glaser BG, Strauss AL (2017). The Discovery of Grounded Theory: Strategies for Qualitative Research.

[43] Neuendorf KA (2017). The Content Analysis Guidebook.

[44] Nicholson Foundation (2019). Over Half of Tweets about ECigs Might Come from Bots.

[45] Allem J, Ferrara E, Uppu SP, Cruz TB, Unger JB (2017). E-cigarette surveillance with social media data: social bots, emerging topics, and trends. JMIR Public Health Surveill.

[46] Varol O, Ferrara E, Davis C, Menczer F, Flammini A (2017). Online human-bot interactions: detection, estimation, and characterization.

[47] Rauchfleisch A, Kaiser J (2020). The false positive problem of automatic bot detection in social science research. PLoS One.

[48] Majmundar A, Moran M (2021). For or against tobacco control: sponsored tobacco advocacy messages on Facebook and Instagram. Nicotine Tob Res.

[49] Bommele J, Hopman P, Walters BH, Geboers C, Croes E, Fong GT, Quah ACK, Willemsen M (2020). The double-edged relationship between COVID-19 stress and smoking: implications for smoking cessation. Tob Induc Dis.

[50] Klemperer EM, West JC, Peasley-Miklus C, Villanti AC (2020). Change in tobacco and electronic cigarette use and motivation to quit in response to COVID-19. Nicotine Tob Res.

[51] Kreslake JM, Simard BJ, O’Connor KM, Patel M, Vallone DM, Hair EC (2021). E-cigarette use among youths and young adults during the COVID-19 pandemic: United States, 2020. Am J Public Health.

[52] Kamiński M, Muth A, Bogdański P (2020). Smoking, vaping, and tobacco industry during COVID-19 pandemic: Twitter data analysis. Cyberpsychol Behav Soc Netw.

[53] Perski O, Simons D, Shahab L, Brown J (2021). Smoking, nicotine and COVID-19: triangulation of methods and pre-registration are required for robust causal inference. Nicotine Tob Res.

[54] Maggi F, Rosellini A, Spezia PG, Focosi D, Macera L, Lai M, Pistello M, de Iure A, Tomino C, Bonassi S, Russo P (2021). Nicotine upregulates ACE2 expression and increases competence for SARS-CoV-2 in human pneumocytes. ERJ Open Res.

[55] Kim E, Ihm J (2020). Online news sharing in the face of mixed audiences: context collapse, homophily, and types of social media. J Broadcast Electron Media.

[56] Center for Countering Digital Hate (2021). The Disinformation Dozen: Why Platforms Must Act on Twelve Leading Online Anti-Vaxxers.

[57] Papacharissi Z (2015). Affective Publics: Sentiment, Technology, and Politics.

[58] Jeffares S (2014). Interpreting Hashtag Politics: Policy Ideas in an Era of Social Media.

[59] Lazer DMJ, Baum MA, Benkler Y, Berinsky AJ, Greenhill KM, Menczer F, Metzger MJ, Nyhan B, Pennycook G, Rothschild D, Schudson M, Sloman SA, Sunstein CR, Thorson EA, Watts DJ, Zittrain JL (2018). The science of fake news. Science.

[60] Shao C, Hui P, Wang L, Jiang X, Flammini A, Menczer F, Ciampaglia GL (2018). Anatomy of an online misinformation network. PLoS One.

