# Supplementary material for: The Influence of Provaping “Gatewatchers” on the Dissemination of COVID-19 Misinformation on Twitter: Analysis of Twitter Discourse Regarding Nicotine and the COVID-19 Pandemic
Source: J Med Internet Res. 2022 Sep 22;24(9):e40331. doi: 10.2196/40331 (PMC9506503; doi:10.2196/40331)
Supplement: Multimedia Appendix 1 [file jmir_v24i9e40331_app1.docx]

**Multimedia Appendix 1. Top 30 search terms identifying COVID-19 tweets.**

|  | | |  |
| --- | --- | --- | --- |
| *covid* | *flattenthecurve* | *staysafe* |  |
| *coronavirus* | “flatten the curve” | “stay safe” |  |
| “corona virus” | *wewillgetthroughthis* | *stayathome* |  |
| “china virus” | “we will get through this” | “stay at home” |  |
| *quarantine* | *stopthespread* | *reopening* |  |
| *isolation* | “stop the spread” | *re-opening* |  |
| *lockdown* | *stayhomesavelives* | *sars* |  |
| *pandemic* | “stay home” | “wuhan virus” |  |
| *epidemic* | “save lives” | “wuhan outbreak” |  |
| **Note:** terms between *s refer to COVID-19-specific hashtags while terms in "s refer to exact text in either the tweet or the metadata of the tweets (e.g. part of a linked URL) | | |  |
|  |  |  |  |
|  |  |  |  |
|  |  |  |  |
